# The Trinity: Interplay among Cancer Cells, Fibroblasts, and Immune Cells in Pancreatic Cancer and Implication of CD8^+^ T Cell-Orientated Therapy

**DOI:** 10.3390/biomedicines10040926

**Published:** 2022-04-18

**Authors:** Yu-Hsuan Hung, Li-Tzong Chen, Wen-Chun Hung

**Affiliations:** 1 National Institute of Cancer Research, National Health Research Institutes, Tainan 704, Taiwan; yhhung@nhri.edu.tw; 2 Division of Hematology & Oncology, Department of Internal Medicine, College of Medicine, Kaohsiung Medical University, Kaohsiung 804, Taiwan; 3 Center for Cancer Research, Kaohsiung Medical University Hospital, Kaohsiung 807, Taiwan

**Keywords:** pancreatic cancer, fibroblasts, immunity, CD8^+^ T cells

## Abstract

The microenvironment in tumors is complicated and is constituted by different cell types and stromal proteins. Among the cell types, the abundance of cancer cells, fibroblasts, and immune cells is high and these cells work as the “Trinity” in promoting tumorigenesis. Although unidirectional or bidirectional crosstalk between two independent cell types has been well characterized, the multi-directional interplays between cancer cells, fibroblasts, and immune cells in vitro and in vivo are still unclear. We summarize recent studies in addressing the interaction of the “Trinity” members in the tumor microenvironment and propose a functional network for how these members communicate with each other. In addition, we discuss the underlying mechanisms mediating the interplay. Moreover, correlations of the alterations in the distribution and functionality of cancer cells, fibroblasts, and immune cells under different circumstances are reviewed. Finally, we point out the future application of CD8^+^ T cell-oriented therapy in the treatment of pancreatic cancer.

## 1. Introduction

Pancreatic cancer is a deadly malignancy around the world according to the latest global reports [1,2] and is expected to be the second leading cause of cancer-related death in 2030 [2]. Surgery [3], radiotherapy [4], chemotherapy [5,6], and immunotherapy [7,8] are available for pancreatic cancer treatment; however, recurrence and metastasis occur [9,10,11,12,13,14,15] frequently after treatment [16]. Therefore, the identification of additional molecular targets and development of novel therapeutic strategies are urgently needed. Pancreatic cancer cells exhibit multiple dysregulations in oncogenes [17,18,19] and tumor suppressor genes [18,20,21,22] to enhance tumor initiation, promotion, and progression. While corresponding targeted therapies work well in some cancer types [23,24,25,26,27], the fibrotic nature of pancreatic cancer makes it hard for drugs to access [28,29]. This property and the immunologically “cold” tumor microenvironment [30,31] render immunotherapy as rarely effective [7,8,32]. Overcoming these barriers may provide a new direction for the design of targeted therapy [33,34,35]. In the pancreatic tumor microenvironment, fibroblasts and immune cells call for the attention of researchers due to their abundance [35,36] and functional diversity [33,34], which we introduce below.

## 2. Fibroblasts and Immune Cells in Pancreatic Cancer

### 2.1. Fibroblast

Fibroblasts in pancreatic cancer can be divided into not only quiescent/activated statuses but also different subtypes, with the latter based on single cell RNA sequencing on patient specimens [37]. The quiescent status of fibroblasts in pancreatic cancer is transformed by external signals [38], leading to morphological change as well as factor secretion such as cytokines, chemokines, and extracellular components [38,39]. The subtype differences in fibroblasts around the pancreas exist before tumor formation [40]. When pancreatic pre-cancerous lesions develop, the fibroblasts surrounding them may receive differential clues due to spatial distribution [35,36,41,42,43,44] and give rise to different phenotypes such as myofibroblast-like, inflammatory, and antigen-presenting. The subtype classifications are further supported by single cell RNA sequencing on mouse pancreatic tumors [45,46]. In three independent single cell RNA sequencing studies [37,45,46], the myofibroblast-like subtype and inflammatory subtype existed in pancreatic bulky tumors, responded to diverse signals, and modulated disease progression differentially. As these subtypes are interchangeable [43,46] and have different prognostic impacts [47], targeting fibroblasts in pancreatic cancer should be considered carefully [35,36,41,42,48,49,50,51], especially with the previous in vivo findings that fibroblast depletion in pancreatic cancer speeded up cancer metastasis [52,53].

Myofibrotic cancer-associated fibroblasts (myCAFs) and inflammatory CAFs (iCAFs) are frequently identified in pancreatic cancer [43,45,46,54,55] and other cancer types [56,57,58,59,60]. In spontaneous pancreatic cancer mouse models, myCAFs and iCAFs exhibit different genetic profiles and oncogenic activities [49]. A myCAF functions in ECM remodeling, expresses α-SMA, localizes near cancer cells, and responds to transforming growth factor β (TGFβ) [36,49,60,61,62,63,64,65,66]. An iCAF acts in factor secretion, expresses interleukin-6 (IL6), localizes distantly from tumor, and responds to IL1 [49,60,62,63,64,65,66]. While an iCAF is tumor-promoting, the comprehensive influence of a myCAF on pancreatic cancer development is still under debate [49,62,65]. What makes CAFs in pancreatic cancer more complicated is their interchangeability [36].

As well as the main CAF subtypes, including myCAF and iCAF, CAF heterogenicity is another important issue in pancreatic cancer development. This heterogenicity starts from the origins of CAFs, which may be resident fibroblasts, pancreatic stellate cells, adipocytes, pericytes, endothelial cells, and mesenchymal stem cells. In terms of subtype heterogenicity, the antigen-presenting CAF (apCAF) [38,67,68] is also present. Common markers for a myCAF include α-SMA [38,40,67] and transgelin [38,40]. For an iCAF, these markers are IL6 [38,40,67] and CXCL12 [38,40]. For the apCAF, the markers are MHCII [40,68] and Saa3 [38,40]. The myCAF in pancreatic cancer associates with increased ECM deposition [69], decreased T cell infiltration [67,68], and altered tumor development [67,68,69]. The iCAF links to increased pancreatic cancer stemness [67], drug resistance [67], tumorigenesis [68,69], and immunosuppression [68,69]. The apCAF mostly associates with antigen presentation [68,69].

### 2.2. Immune Cell

The immune cell is another critical component in the pancreatic cancer microenvironment [29,33,70,71,72,73,74,75,76,77]; though, with its immunologically cold nature partially attributed to desmoplasia, pancreatic cancer patients with CD8^+^ lymphocyte infiltration have a better prognosis [78,79]. For other lymphoid cells, the role of CD4^+^ lymphocytes in pancreatic cancer heavily depends on the helper cell type. The existence of FoxP3^+^ regulatory T lymphocytes (T_reg_) are associated with a higher risk for pancreatic cancer [78]. Conversely, the amount of B lymphocytes predicts better pancreatic cancer prognosis [78,79]. The presence of nature killer (NK) cells also benefits patient survival [70] as long as their cytotoxicity is retained [70,77,79]. In myeloid cells, mast cells associate with pancreatic cancer growth and angiogenesis [80,81,82]. A higher ratio of neutrophils/lymphocytes links to a poor clinical outcome of pancreatic cancer patients in meta-analyses [83,84]. On the contrary, macrophages affect pancreatic cancer cell behaviors in a polarization-dependent manner. M2 macrophages, with antiinflammatory property, frequently block immune surveillance and promote cancer growth. In contrast, M1 macrophages with pro-inflammatory character elicit immunity to fight against tumor. However, dissection of tumor-associated macrophages (TAM) in pancreatic cancer revealed that these macrophages may display features of both polarization types. These phenomena were widely observed and reviewed in detail [34,71,73,76,79,85]. While the mechanism by which dendritic cells influence pancreatic tumors is still unclear, its potential anticancer efficiency via vaccination has been observed for a long time [86,87,88,89,90,91] and has been recently revisited [92,93,94].

In addition to the M2 macrophage, myeloid-derived suppressor cells (MDSC) play an important role in pancreatic tumorigenesis [95]. These suppressor cells include monocytic (M-MDSC) and polymorphonuclear cells (PMN-MDSC). They arise from the impaired differentiation of myeloid progenitor cells during myelopoiesis, and this impairment is commonly observed during tumorigenesis [96]. For M-MDSC, Trovato et al. found both M-MDSC and PMN-MDSC in pancreatic cancer tissues, negatively correlated with T cell population [97]. In the peripheral blood mononuclear cell (PBMC) subset, M-MDSC percentage was higher in pancreatic cancer patients than in healthy donors. Using whole blood samples in the validation cohort, the authors found CD14^+^ M-MDSC still displayed greater suppression on T cell proliferation than CD66b^+^ PMN-MDSC did. This resulted from STAT3 activation and subsequent arginase expression, and could be counteracted by arginase inhibition. For PMN-MDSC, Peng et al. reported that, in an H7 orthotopic pancreatic cancer mouse model, apolipoprotein A-I mimetic peptide L-4F decreased tumorigenesis via PMN-MDSC suppression [98]. Increments in CD8^+^ T cells and IFNγ in the tumor and spleen after L-4F treatment were observed, compared to a control Sc-4F treatment group. Mechanistically, this suppression was achieved by inhibitions of ROS production and STAT3 phosphorylation. Conversely, Kramer et al. showed that PMN-MDSC in pancreatic cancer was associated with the increased expression of Wnt inhibitor Dickkopf-1 (Dkk1) [99].

## 3. Mechanisms Underlying the Interplay among Pancreatic Cancer Cells, Fibroblasts, and Immune Cells

### 3.1. Cytokine and Chemokine

**Figure 1:**
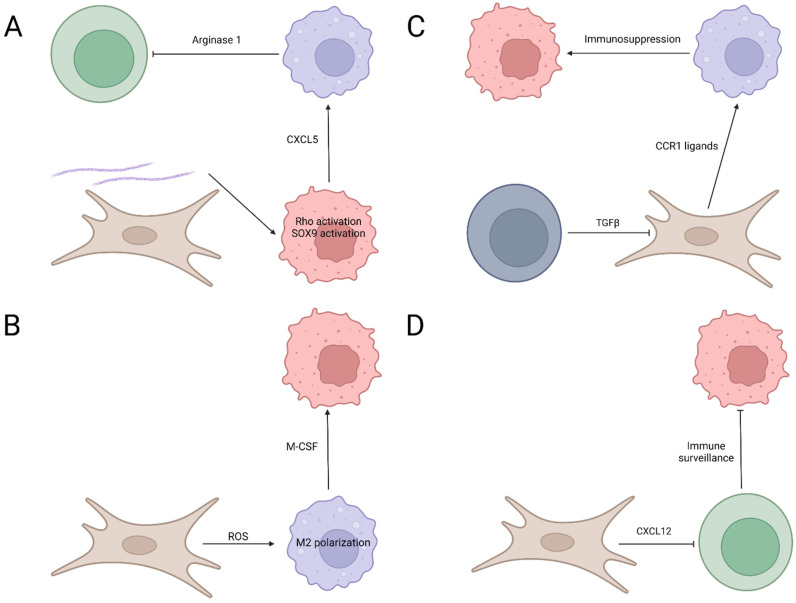
Mechanisms underlying the interplay among pancreatic cancer cells, fibroblasts, and immune cells. (**A**) Collagen I around fibroblasts (brown) stimulates pancreatic cancer cells (red) and increases the Rho/SOX9 activation, which leads to CXCL5 expression to activate myeloid cells (purple). Myeloid cells, in turn, express arginase 1 to suppress CD8^+^ T cell (green). (**B**) Fibroblasts (brown) modulate macrophage (purple) polarization and function via ROS, and M2 polarized macrophages assist the proliferation of pancreatic cancer cell (red) via macrophage colony-stimulating factor (M-CSF). (**C**) T_reg_ (gray) express TGFβ to stimulate the expression of CCR1 ligands in fibroblasts (brown), and the CCR1 ligands recruit myeloid cells (purple) to promote the growth of pancreatic cancer cells (red). (**D**) Fibroblasts (brown) express CXCL12 to suppress CD8^+^ T cells (green) and block immune surveillance against pancreatic cancer cells (red).

What makes the situation even more complicated is the interplay among pancreatic cancer cells, fibroblasts, and immune cells. A change in one component affects the whole picture in a pancreatic tumor [33,34,35,36,48,50,51,70,73,74,75,77,100]. Mechanistically, cytokines and chemokines secreted from pancreatic cancer cells, fibroblasts, or immune cells modulate the behaviors of the other two populations [35,36,49]. However, only limited targets have been identified in these multi-directional interplays [101] (Table 1 and Figure 1). Mucciolo et al. utilized knockout mice and antibody treatment to explore the role of IL17 in the pancreatic tumor microenvironment and found the loss of this cytokine modulated extracellular matrix (ECM) composition and increased CD3^+^ cell infiltration [101]. In pancreatic cancer cells, mutant Kras elicited IL17 expression [101,102], possibly via the STAT3 signaling pathway [103,104]. IL17, in turn, worked on fibroblasts to reshape the ECM [101,105] and on immune cells to block their antitumor function [106,107,108]. With a similar approach, Zhang et al. revealed that regulatory T cells’ (T_reg_) depletion in a transgenic pancreatic cancer mouse model surprisingly accelerated pancreatic tumorigenesis via inactivation of the PDGFRβ^+^ SMA^+^ myofibroblast [109]. In addition, compensation by other pro-tumor myeloid-derived suppressor cells (MDSC) was observed once T_reg_ were depleted. The results of RNA sequencing pointed out that in this process, many C-C chemokine receptor type 1 (CCR1) ligands were upregulated, and CCR1 inhibition retarded pancreatic tumorigenesis, resulting from T_reg_ depletion. In terms of fibroblasts, Feig et al. showed that fibroblast activation of protein-positive (FAP)^+^ cancer-associated fibroblasts (CAFs) participated in pancreatic cancer formation, and the depletion of FAP^+^ fibroblasts in a transgenic pancreatic cancer mouse model decreased tumor development [110]. This might be through C-X-C motif chemokine ligand 12 (CXCL12) expression in FAP^+^ fibroblasts and its effect on T cell exclusion, specifically via high mobility group box 1 (HMGB1) [111,112]. Accordingly, loss of the CD4/CD8 cells reversed the effect of FAP^+^ fibroblast depletion on tumor growth. In addition, the inhibition of CXCR4, the cognate receptor for CXCL12, by AMD3100 improved anti-PD-L1 efficiency.

### 3.2. Extracellular Signal

In addition to cytokines and chemokines, molecules such as extracellular components and stress signals also participate [113,114] in the interaction among the “Trinity”. Chen et al. demonstrated that the loss of collagen I in α-SMA^+^ fibroblasts enhanced pancreatic cancer progression via CXCL5 upregulation in cancer cells and the subsequent activation of MDSC [113]. As CXCL5 is one of the most upregulated factors in a pancreatic cancer mouse model with collagen I knockout, the authors proposed that this ECM alteration stimulated SOX9 transcription activation [113,115] in cancer cells [113]. CXCL5 then recruited and polarized MDSC into a pro-tumor type to enhance the growth of pancreatic cancer cells. Furthermore, MDSC suppressed functions of T and B cells via arginine depletion. These effects on ECM alteration and immune suppression via cancer cells and MDSC emphasized the importance of α-SMA^+^ fibroblasts in pancreatic tumorigenesis by affecting collagen deposition. In terms of stress signals, Zhang et al. established primary cultures of fibroblasts from human pancreatic cancer tissues, and found they affected monocytes in a reactive oxygen species (ROS)-dependent manner [114]. With either a CAF-conditioned medium or direct CAF co-culture, monocytes displayed increased M2 polarization. This phenomenon was associated with upregulated ROS production in monocytes, and both ROS amount and M2 polarization were inhibited by antioxidants. CAFs increased ROS in monocytes via M-CSF, which is specific to cancer-associated fibroblasts as normal fibroblasts rarely produce this factor. Moreover, the CAF-stimulated monocytes are capable of inducing Stat3 and Akt phosphorylation in pancreatic cancer cells and subsequently increasing their growth. Pancreatic cancer cells also express a variety of factors including VEGF [116] to activate Stat3 and Akt [117,118], and subsequently modulate macrophage behavior. This study suggests the complex interaction among cancer cells, fibroblasts, and immune cells during pancreatic cancer development.

### 3.3. Pathway Modulation

Researchers also utilized additional approaches to dissect the interplay among the “Trinity” members in pancreatic tumors, such as inhibitor treatment and gene delivery. Zhao et al. applied M-CPA/PTX, a polymeric micelle-based nanoformulation of sonic hedgehog inhibitor (cyclopamine, CPA) and paclitaxel (PTX), to modulate stroma in pancreatic cancer [119]. M-CPA/PTX increased intratumoral vasculature density to benefit CD8^+^ T cell infiltration without altering the ECM and fibroblasts. In the bulky tumor, VEGFR2 and Shh phosphorylations were suppressed by M-CPA/PTX. Moreover, M-CPA/PTX improved anti-PD-1 efficiency via IFN-γ from CD8^+^ T cells. Zhou et al. directly applied gene delivery targeting relaxin to hamper fibrosis in cancers [120]. Relaxin is an insulin-like peptide hormone with diverse biological functions but is known for fibrosis resolution therapeutically [120,121]. The authors utilized this advantage to test its effect on pancreatic desmoplasia by targeting macrophages as they express the relaxin receptor (relaxin family peptide receptor type 1, RXFP1). They found that local relaxin plasmid (pRLN) delivery in the format of aminoethyl anisamide (AEAA)-conjugated lipid-protamine-DNA (LPD) nanoparticle induced macrophage recruitment [121]. These macrophages uptook relaxin and activated themselves to express cytokines and enzymes for fibrosis depletion. This phenomenon is accompanied with (1) a decrease in α-SMA^+^ fibroblasts; (2) a reduction in collagen I; (3) an increase in antitumor CD8^+^ T and NK cells; (4) attenuation in pro-tumor T_reg_. Furthermore, pRLN benefited anti-PD-L1 efficiency, even though pRLN itself already displayed a better treatment effect in vivo. Although the advantage of stroma targeting in pancreatic cancer is still under debate [52,53], the works from Zhao et al. [119] and Zhou et al. [120] supported the notion that the entry of CD8^+^ T cells has a higher benefit than the depletion of fibroblasts in this process, as CD8^+^ T cell infiltration is the main determinant of treatment efficiency with or without fibroblast alteration.

## 4. Correlation of Alterations in the “Trinity” Population in Preclinical Model and Clinical Setting

In addition to discussing the mechanisms of the multi-functional interplay between cancer cells, fibroblasts, and immune cells, we also summarized the correlation of alterations of the “Trinity” members under different circumstances (Table 2). Pancreatic tumor tissues showed increased focal adhesion kinase (FAK) activation and ECM accumulation. Jiang et al. reported that the FAK inhibitor accordingly reversed ECM alteration and suppressed tumorigenesis in animals. The authors demonstrated that MDSC infiltration was suppressed by the FAK inhibitor. In this preclinical model, co-reduction in fibroblasts and MDSCs was found after FAK inhibition, suggesting a role of FAK in controlling the population of these two cell types in pancreatic cancer [122].

Gorchs et al. isolated fibroblasts from human pancreatic tumors and found that CAFs expressed more PD-L1 and PD-L2 compared to normal skin fibroblast [123]. The co-culture of CAFs resulted in the suppression of T cell proliferation in a prostaglandin E2 (PGE2)-dependent manner. Consequently, PGE2 inhibition by the COX2 inhibitor restored this phenotypic change. For those T cells still proliferating, CAFs also hindered their function by (1) increasing co-inhibitory molecules and (2) decreasing functionality, suggesting the antagonism of cell number and biological activity between fibroblasts and T cells.

Ireland et al. showed stromal Gas6 inhibition by neutralizing antibody suppressed metastasis in a spontaneous pancreatic cancer mouse model [124]. To find the origin of Gas6 secretion, the authors used a cell sorting approach and confirmed macrophages and fibroblasts were the main Gas6 producers. Additionally, immunohistochemical (IHC) staining on serial sections further verified the activation of downstream AXL signaling [125,126]. A Gas6 blockade decreased the epithelial–mesenchymal transition (EMT) in pancreatic cancer cells, while neither myeloid cells nor T cells were affected. However, Gas6 inhibition reduced NK cell recruitment. These results suggest that fibroblasts and macrophages modulated cancer cells in a unidirectional way via Gas6.

The correlation of the ECM and immune cells in clinical samples has also been investigated. Kamionka et al. showed the collagen composition in pancreatic tumor tissues was not significantly linked with immune cell infiltration, suggesting collagen organization has a minor impact on T cell distribution [127].

To understand the associations of different cell types in the tumor microenvironment, Lawal et al. applied databases to analyze multi-omics results of multiple cancers emphasizing the STAT3/CDK2/4/6 axis [128]. As this axis is highly expressed across various cancer types, the authors correlated it to epigenetics and immunity, and found an association between CAFs and STAT3 in pancreatic cancer. With a similar high-throughput concept, MacNeil et al. surveyed the prognostic impact of CAFs and tumor-infiltrating lymphocytes (TIL) in pancreatic cancer patients [129]. Multivariate analysis showed that CD8^+^ T cells of TIL and FAP^+^ CAF are predictors of survival; the former predicted better survival, while the latter correlated with worse survival. On the other hand, Tahkola et al. directly analyzed the effect of hyaluronan accumulation on pancreatic cancer immune cell infiltration, and found the infiltration was significantly interrupted by hyaluronan as reflected by a low immune cell score across survival types (overall and disease-specific) via univariate and multivariate analyses [130]. Moreover, Nizri et al. studied pancreatic cancer lymph node (LN) metastasis and analyzed its association to desmoplasia and the ECM profile. They found collagen, TGFβ (fibroblast-related), and GATA3 ((Th2) immunity-related) are expressed mainly in desmoplasia-high LN [131]. A similar association in pancreatic cancer was also observed in the study by Sadozai et al. [132]. The authors analyzed the components in the pancreatic tumor microenvironment by IHC staining and RNA sequencing to compare the differences in long-term and short-term survivors. The results from both studies revealed that (1) a long-term survivor has less α-SMA and (2) a long-term survivor has more CD3^+^ T cells in the tumor and stroma parts. State-of-the-art technology such as single cell RNA sequencing accelerates target/solution identification in cancer research, and with this approach, Wang et al. identified the CAF subtype with a highly activated metabolic state (meCAF) in pancreatic cancer patients with loose stroma (low desmoplasia) [55]. Under this circumstance, CD8^+^ T cells are more abundant in both single cell RNA sequencing and IHC analysis. While the meCAF predicted worse survival, it displayed a better response toward anti-PD-1. This work suggested the identification and potential application of a novel fibroblast subtype for pancreatic cancer research and therapy. Collectively, the aforementioned studies conclude that the alterations in the “Trinity” members in pancreatic tumors provide useful information in predicting a patient’s clinical outcome and in providing a therapeutic strategy.

## 5. Treating Pancreatic Cancer with CD8^+^ T Cell-Orientated Approach

### 5.1. Preclinical Study

**Figure 2:**
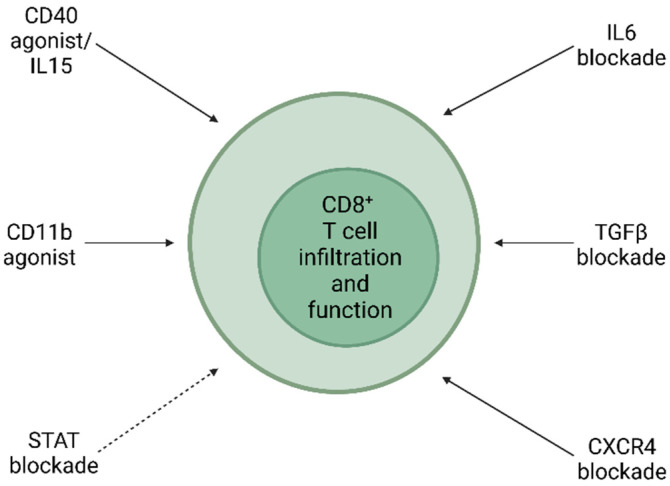
Treating pancreatic cancer with CD8^+^ T cell-orientated approaches. Potential CD8^+^ T cell-orientated treatments for pancreatic cancer were reviewed and blockade of IL6/TGFβ/CXCR4/STAT and activation of CD40/IL15/CD11b were proposed to be effective in pancreatic cancer therapy. Solid arrow represents direct effect, while dotted arrow represents indirect effect.

Treating pancreatic cancer by directly increasing CD8^+^ T cell infiltration and/or function is a new idea with clinical merit (Figure 2). Mace et al. found that an IL6 blockade improved anti-PD-L1 efficiency and retarded pancreatic tumorigenesis [133]. With the IL6 expression by pancreatic stellate cell (PSC) in mind, the authors tested the effect of an IL6 neutralizing antibody on subcutaneous tumors of MT5 and Panc02 (mouse pancreatic cancer cell lines), and demonstrated that while IL6 antibody itself had a minor effect, its combination benefited anti-PD-L1 efficiency. This is correlated to increased effector CD8^+^ T cell infiltration in the tumors. On the other hand, only CD8^+^ cell neutralization, but not CD4^+^ cell neutralization, blunted the antitumor effect that resulted from the IL6 blockade. In an orthotopic model, treatment of the IL6 antibody could not prevent CD3^+^ cell recruitment. Principe et al. demonstrated that TGFβ is of equal importance in this kind of treatment, as KC mouse (P48-Cre × LSL-KRAS^G12D^) receiving wild-type CD8^+^ T cell still displayed disease progression, but tumor growth was ceased when the CD8^+^ T cells harvested from Tgfbr1^+/−^ mice were injected [134]. Consistent with this finding, TGFβ inhibitor galunisertib also showed the same inhibitory effect in the animal model and displayed better efficiency when combined with anti-PD-1 antibody. While CD8^+^ T cell infiltration was not increased, its functions regrading memory subset and IFNγ/perforin expression were all boosted. In addition to IL6 and TGFβ, CXCR4-mediated signaling also affects pancreatic cancer treatment significantly. Two studies addressed the importance of CXCR4 in pancreatic tumorigenesis [135,136]. Seo et al. applied patient specimens to investigate the influence of CXCR4 inhibitor AMD3100 on apoptosis, and found it did so by redistributing the CD8^+^ T cells to the juxtatumoral stroma with the potential release of granzyme B and IFNγ. In the COMBAT clinical trial, Bockorny et al. applied another CXCR4 antagonist, BL-8040, to test its antitumor effect together with pembrolizumab for pancreatic cancer patients. Patients receiving combinatory therapy displayed increased CD8^+^ T cell mobilization, especially that of cytotoxic (granzyme B^+^) CD8^+^ T cells. When further combined with chemotherapy, most patients displayed a better response compared to their own baselines.

In addition to cytokine/chemokine, the direct activation of CD8^+^ T cells by membrane proteins is also implicated in pancreatic cancer treatment. Audenaerde et al. and Panni et al. investigated the role of CD40 agonist/IL15 and CD11b agonist, respectively, for this purpose [137,138]. Audenaerde et al. injected mouse cancer cells subcutaneously and tested the effect of different molecules on tumor growth. They found that CD40 agonist/IL15 is the most effective for suppressing tumor growth. This was achieved by the CD8^+^ T cell as its depletion reversed the therapeutic effect. Accompanying this phenomenon, antitumor factors and effector/memory subsets were all increased. Moreover, the suppressive effect of CD40 agonist/IL15 could still be observed in re-challenge experiments after a 100-day tumor-free period. Panni et al., alternatively, utilized CD11b agonist ADH-503 on an orthotopic mouse model and demonstrated this agonist promoted the recruitment of immune cells including cytotoxic and memory CD8^+^ T cells under rapid proliferation and activated their antitumor functions. Mechanistically, ADH-503 acted through a conventional dendritic cell (cDC) to achieve tumor suppression, as demonstrated by the lack of response to ADH-503 in a cDC-lacking Batf3^−/−^ mouse. Impressively, the CD11b agonist itself displayed significant efficiency in pancreatic cancer treatment as a sole agent in both subcutaneous and orthotopic models. More importantly, ADH-503 combined with chemotherapy and immunotherapy to dramatically inhibit tumor growth. Modulation of the immune microenvironment is equally important. Michaelis et al. demonstrated that TLR7/8 agonist R848 prolonged survival in intraperitoneal and orthotopic mouse models of pancreatic cancer [139]. R848 itself has been shown to decrease tumor mass via modulations on the immune microenvironment and cachexia.

Finally, the questions raised are how is CD8^+^ T cell-oriented therapy applied to pancreatic cancer treatment and does the entry of T cells modulate other immune cells to create a favorable anticancer immunity? Ajina et al. showed that the T cell is the determinant for pancreatic cancer suppression in various mouse strains and is a critical factor affecting the recruitment of myeloid cells [140]. They applied NanoString analysis and found Stat1 in a pancreatic cancer cell was upregulated when a T cell was presented. The upregulation of Stat1, in turn, activated the myeloid cell to blunt the T cell’s antitumor effect. As a result, FDA-approved Stat inhibitor ruxolitinib further improved anti-PD-L1 efficiency in the aforementioned models, although ruxolitinib itself displayed minor efficiency. While this improvement on pancreatic cancer suppression may not solely result from myeloid cell inhibition, as it was suggested by the authors that the abundance of MDSC was not significantly decreased, this study pinpointed the importance of CD8^+^ T cell-orientated treatment [141].

### 5.2. Clinical Trial

In validation of the results of the preclinical studies described above, we reviewed related clinical trials in the database (https://www.clinicaltrials.gov/ (accessed on 28 February 2022). For pancreatic cancer, STAT inhibitor was applied in trials and the benefits of this agent were reported (NCT02993731) [142] (Table 3). In this randomized, open-label, multi-center, phase 3 study, the effect of napabucasin (2-acetylfuro-1,4-naphthoquinone, BBI-608), a STAT3 inhibitor, on metastatic pancreatic ductal adenocarcinoma was investigated. The effects on disease progression of 1134 patients treated with napabucasin + nab-paclitaxel or nab-paclitaxel + gemcitabine were compared. In the napabucasin + nab-paclitaxel arm, patients were given napabucasin twice daily and the other two agents weekly. In arm 2 of nab-paclitaxel + gemcitabine, patients were given nab-paclitaxel and gemcitabine with the same frequency. The primary outcome was overall survival, and the secondary outcomes were progression-free survival, overall response rate, adverse events, and quality of life. No difference in overall survival was found between the two groups’ 565 patients (11.43 vs. 11.73 months in arms 1 and 2, respectively. For secondary outcomes, the progression-free survival in arm 1 and 2 was 6.70 vs. 6.08 months, respectively; disease control rate was 74 vs. 76%, respectively; overall response rate in both arms was 43%; adverse events was 99.8% vs. 99.3%, respectively; quality of life score was −1.63 versus −0.57, respectively (higher score represents a higher quality of life). Although the combination of napabucasin and nab-paclitaxel does not show a better effect than nab-paclitaxel + gemcitabine, this study helps to define how this STAT3 inhibitor influences disease progression in phospho-STAT3-positive patients and its potential application in the future. Recently, the effects of napabucasin in clinical trials for multiple cancers have been updated [142,143,144].

Although the benefits of targeting IL6, TGFβ, CXCR4, and CD40 in pancreatic cancer remain elusive, clinical trials for these factors in other cancer types have shown promise. The effects of the anti-IL6 antibody in treating multiple myeloma were tested in NCT00911859 and NCT01484275 trials. In NCT00911859, this randomized, open-label, phase 2 study investigated an IL6 antibody (CNTO 328, siltuximab) and its combination with velcade-melphalan-prednisone (VMP) to treat multiple myeloma [145]. The safety of siltuximab (11 mg/kg as a 1-h intravenous infusion every 3 weeks) in patients was first confirmed in part 1 of this trial. In part 2, treatment efficiency was compared between arm A of VMP + siltuximab and arm B of VMP. The primary outcome was complete response, and secondary outcomes were overall response, duration of response, progression-free survival, overall survival, and quality of life. In arm A (52 patients) and arm B (54 patients), complete response was 26.5 and 22.4%, respectively. For secondary outcomes, in arm A and arm B, the overall response was 87.8 versus 79.6%, respectively; duration of response was 583 versus 497 days, respectively; progression-free survival was 519 versus 518 days, respectively. Conversely, the quality of life score was 8.33 and 14.78 for arm A and B, respectively. In a related publication [145], San-Miguel et al. further showed that (1) the partial response in arm A and arm B was 71 and 51%, respectively (*p* = 0.0382); (2) adverse event in arm A and arm B was 92 and 81%, respectively (*p* = 0.09). The authors concluded that there is no significant improvement in terms of complete response and outcome using siltuximab among patients with multiple myeloma. In NCT01484275, siltuximab was applied to test its safety and efficiency for patients with high-risk smoldering multiple myeloma (SMM). The safety of siltuximab in SMM patients was confirmed (15 mg/kg as a 1-h intravenous infusion every 4 weeks). This randomized, double-blind, placebo-controlled, multi-center, phase 2 trial enrolled 85 patients. The siltuximab arm (43 patients) and placebo arm (42 patients) had one-year progression-free survival rates of 84.5 and 74.4%, respectively. The time to disease progression was 125.5 versus 118 days in the siltuximab and placebo group, respectively. Therefore, siltuximab does not improve the survival of SMM patients [146].

For CXCR4, two trials (NCT00906945 and NCT00512252) studied the impact of CXCR4 blockage on disease inhibition in acute myeloid leukemia (AML). In the NCT00906945 study, the applications of G-CSF as well as CXCR4 blockade (plerixafor, AMD3100) were performed to investigate the clinical benefits. This non-randomized, open-label, phase 1/2 trial enrolled 39 patients. The results of this study showed a complete response rate of 30% and suggested the benefit of CXCR4 inhibition in AML patients [147]. In the NCT00512252 trial, without G-CSF, AMD3100 still increased the effectiveness of mitoxantrone, etoposide, and cytarabine (MEC) in AML patients. It was concluded that the CXCR4 inhibitor mobilized the AML cell to circulation and improved treatment efficiency, and plerixafor could be safely combined with chemotherapy to treat relapsed or refractory AML [148].

For membrane protein activation and its related cancer treatment, targeting CD40 was investigated in NCT00101166 and NCT01433172 trials. These trials applied a cancer vaccine that is composed of tumor cells from patient and bystander cells expressing GM-CSF and CD40L. These cells were treated with high-dose X-rays to ensure the loss of oncogenicity. This vaccine is able to better boost immunity in patients. In the NCT00101166 trial, the investigators reported that the recruitment of the CD1a^+^CD86^+^ dendritic cell could be observed in the injection site via immunohistochemistry analysis [149]. In addition, the cancer vaccine worked well for several types of solid tumors. In the NCT01433172 study, in addition to the cancer vaccine mentioned above, cytokine CCL21 was added to assist T cell recruitment and responsiveness. The results of this study revealed that GM-CSF + CD40L + CCL21 vaccination induced abundant tumor-infiltrating lymphocytes in tumors, which suggested the benefit of CD40 blockade in cancer treatment [150]. Taken together, these clinical trials shed light on the importance of IL6, TGFβ, CXCR4, and CD40 in cancer treatment, as well as their potential therapeutic effects on pancreatic cancer. Even though these clinical trials were applied to patients with cancer types other than pancreatic cancer, and the hazard ratio as well as statistical significance may be restricted due to the enrolled patient numbers, they suggest the potential application of the above treatments for pancreatic cancer patients.

### 5.3. Clinical Correlation

**Figure 3:**
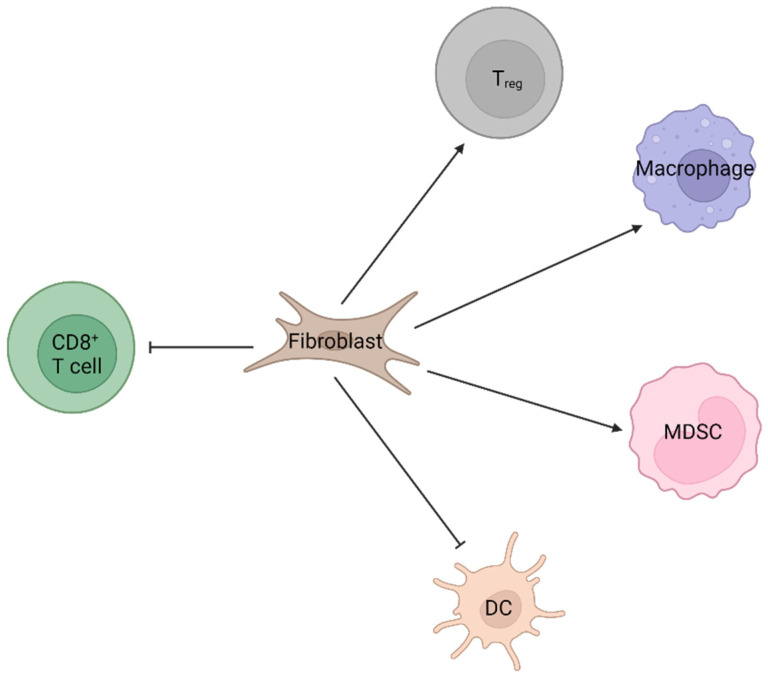
Scheme shows the potential effects of fibroblast on immune cell recruitment/function in pancreatic cancer. Based on the findings of recent studies, the potential effect of fibroblast on recruitment and function of CD8^+^ T cell, Treg, macrophage, MDSC, and DC in pancreatic cancer is proposed.

In addition to macrophages and neutrophils, the dendritic cell (DC) adds an important portion to cancer immunity via antigen presentation. The dendritic cell in pancreatic cancer also correlated with better survival [151]. Conventional DC type 1 (cDC1), type 2 (cDC2), and plasmacytoid DC (pDC) were enriched more in the stroma than in the tumor. cDC1 in the tumor stroma further predicted better disease-free survival (DFS). Mechanistically, DC function in pancreatic cancer is affected by multiple factors and proteins. Pancreatic cancer cell-derived prostaglandin E2 (PGE2) and regenerating islet-derived protein 3A (Reg3A) decreased DC function via cellular stresses and JAK2 modulation, respectively [152,153]. This subsequently led to a decrease in T cell proliferation [153]. The stromal protein also influenced DC effectiveness during pancreatic tumorigenesis as Giri et al. reported that the loss of heat shock protein 70 (Hsp70) in DC increased its functionality [154]. In terms of pancreatic cancer treatment, DC is also a critical target. Sadeghlar et al. showed that CD40L transduction in human DC increased its function and proliferation, even in the presence of a pancreatic cancer cell in co-culture [155], and Kim et al. reported that cetuximab-conjugated maleimide-polyethylene glycol-chlorin e6 induced pancreatic cancer cell death, which was accompanied by DC activation and maturation [156]. Salah et al. demonstrated that pulsed DC increased antigen-specific cytotoxic T cells among pancreatic cancer patients [157]. Recently, Kvedaraite et al. also summarized that pancreatic cancer patients had fewer DC progenitors and cDC1s, and pDC predicted better prognosis [158] (Figure 3).

Predictive signatures are essential for improvements in pancreatic cancer diagnosis and treatment. With databases including The Cancer Genome Atlas (TCGA), 2-gene TP53-associated immune prognostic model (TIPM) [159], 15-gene immune, stromal, and proliferation-associated (ISP) signature [160], 47-gene immune-related gene pairs (IRGPs) [161], 8-gene signature [162], 59-gene TMEscore-high group [163], and 5-gene module immune cluster [164], we could predict pancreatic cancer prognosis more precisely. At protein level with tissue arrays, signatures of leukocyte subtype/stromal composition [165] also predicted differential survival among pancreatic cancer patients. Santiago et al. reviewed immunophenotypes in pancreatic cancer in great detail [166].

To sum up, with all the studies and reviews above, it is clear that the complex nature of the interplay between pancreatic cancer cells, fibroblasts, and immune cells is a key factor determining tumor formation and treatment response. In addition to therapies targeting fibroblasts [35,36] and immune cells [71,72], how these cells affect the rest of the bulky tumor to create a tumor-promoting microenvironment is a critical issue for continuous exploration. Both hypothesis-driven study [113,120] and high-throughput analysis [45,55] will strengthen our understanding of this important interplay guided by the cancer cells, stroma, and immunity in pancreatic cancer [140,167].

## Figures and Tables

**Table 1:** Mechanisms underlying the interplay among pancreatic cancer cells, fibroblasts, and immune cells.

Molecule	Cell Type	Mechanism	Reference
TGFβ	T_reg_	TGFβ from T_reg_ activates SMA^+^ myofibroblasts and their expression of CCR1 ligands to recruit MDSC	[109]
CXCL12	CAF	CXCL12 from FAP^+^ CAF increases T cell exclusion and cancer growth	[110]
Type I collagen	Myofibroblast	Type I collagen from myofibroblast decreases SOX9 expression and subsequently increases CXCL5 in PDAC to recruit MDSC	[113]
ROS	CAF	ROS from CAF increases monocyte differentiation into M2 macrophages and their production of M-CSF to increase the invasiveness of PDAC	[114]

**Table 2:** Correlation of the alterations in cancer cells, fibroblasts, and immune cells in co-culture, animal models, and clinical samples.

Molecule	Cell Type	Association		Reference
NA	PDAC	Collagen/fibroblast	↓ post FAKi treatment	[122]
		Macrophage	↓ post FAKi treatment	
		G-MDSC	↓ post FAKi treatment	
NA	PDAC	CAF	PD-L1/PD-L2↑	[123]
			CD4/CD8 proliferation ↓	
			CD4/CD8 co-inhibitory marker↑	
			CD8 function ↓	
NA	PDAC	Vimentin	↓ post α-Gas6 treatment	[124]
		NK	↓ post α-Gas6 treatment	
NA	PDAC	Collagen	Density not altered	[127]
		T cell	Infiltration not altered	
CDK2/4/6	PDAC	CAF	Co-occurrence ↑	[128]
STAT3		CAF	Co-occurrence ↑	
		Immunity	Onco-immune signature ↑	
NA	PDAC	CD4	Disease progression ↓	[129]
		CD8	Disease progression ↓	
		Thy-1^+^ CAF	Disease progression ↓	
		FAP^+^ CAF	Disease progression ↑	
Stromal hyaluronan accumulation	NA	CD8/CD3-based immune cell score	↓	[130]
NA	PDAC	Desmoplasia	COL11A1/COL11A2/COL1A1/TGF-β mRNA ↑	[131]
		Th2 immunity	GATA3 ↑	
NA	PDAC	α-SMA/fibrosis	↑ in STS	[132]
NA	PDAC	CD68/CD163	↑ in STS	
		CD4	↓ in STS	
		iNOS	↓ in STS	
		Foxp3	↑ in STS	
		B cell/DC	↓ in STS	
NA	PDAC	Metabolic active CAF (meCAF)	↑ in dense (high desmoplasia) group	[55]
		CD8	↑ in loose (low desmoplasia) group	
		Response to α-PD-1	↑ in loose group	

FAK, focal adhesion kinase; STS, short-term survivors; ↑, increment; ↓, decrement.

**Table 3:** Summary of clinical trials targeting IL6, TGFβ, CXCR4, CD40, and STAT for cancer treatment.

Clinical Trial ID	Treatment	Cancer Type	Reference
NCT02993731	Napabucasin + nab-paclitaxel(+gemcitabine)	Pancreas	[142]
NCT00911859	Siltuximab(+velcade-melphalan-prednisone)	Multiple myeloma	[145]
NCT01484275	Siltuximab	Smoldering multiple myeloma	[146]
NCT00906945	G-CSF + plerixafor + mitoxantrone + etoposide + cytarabine	Acute myeloid leukemia	[147]
NCT00512252	plerixafor + mitoxantrone + etoposide + cytarabine	Acute myeloid leukemia	[148]
NCT00101166	GM.CD40L vaccination	Melanoma	[149]
NCT01433172	GM.CD40L vaccination (+CCL21)	Lung	[150]

Napabucasin, STAT3 inhibitor; siltuximab, IL6 antibody; plerixafor, CXCR4 inhibitor.
